# Supplementary material for: Behavior choices amongst grooming, feeding and courting in Drosophila show contextual flexibility, not an absolute hierarchy of needs
Source: J Exp Biol. 2025 Nov 28;228(23):jeb250826. doi: 10.1242/jeb.250826 (PMC12745933; doi:10.1242/jeb.250826)
Supplement: Supplementary information [file jexbio-228-250826-s1.pdf]

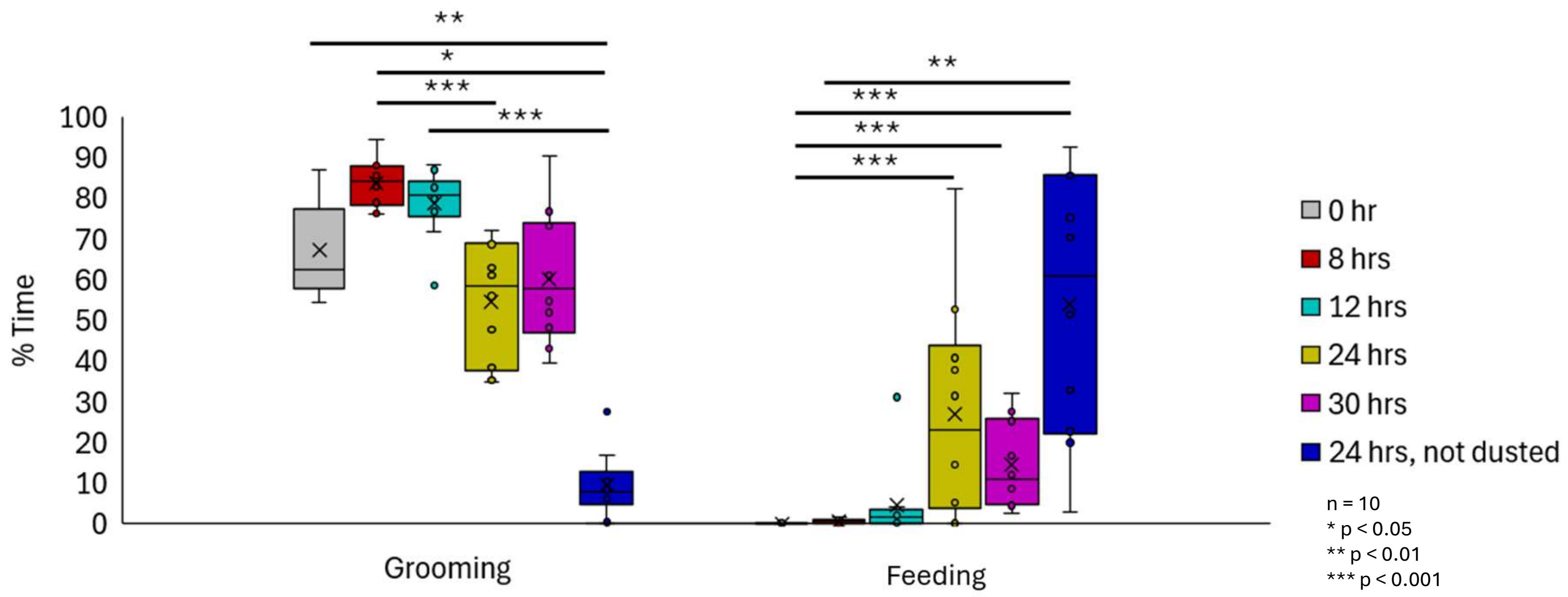

Fig. S1.*CantonS* behavior changes with respect to hunger drive.

Box plot depicting the total percent time spent grooming and feeding during a grooming vs feeding assay at variable levels of starvation. X denotes mean, bar denotes median, whiskers denote the lower and upper quartile. Statsiticas were obtained using a Kruskal-Wallis test followed by a Dunn's multiple comparison test and Bonferroni correction.

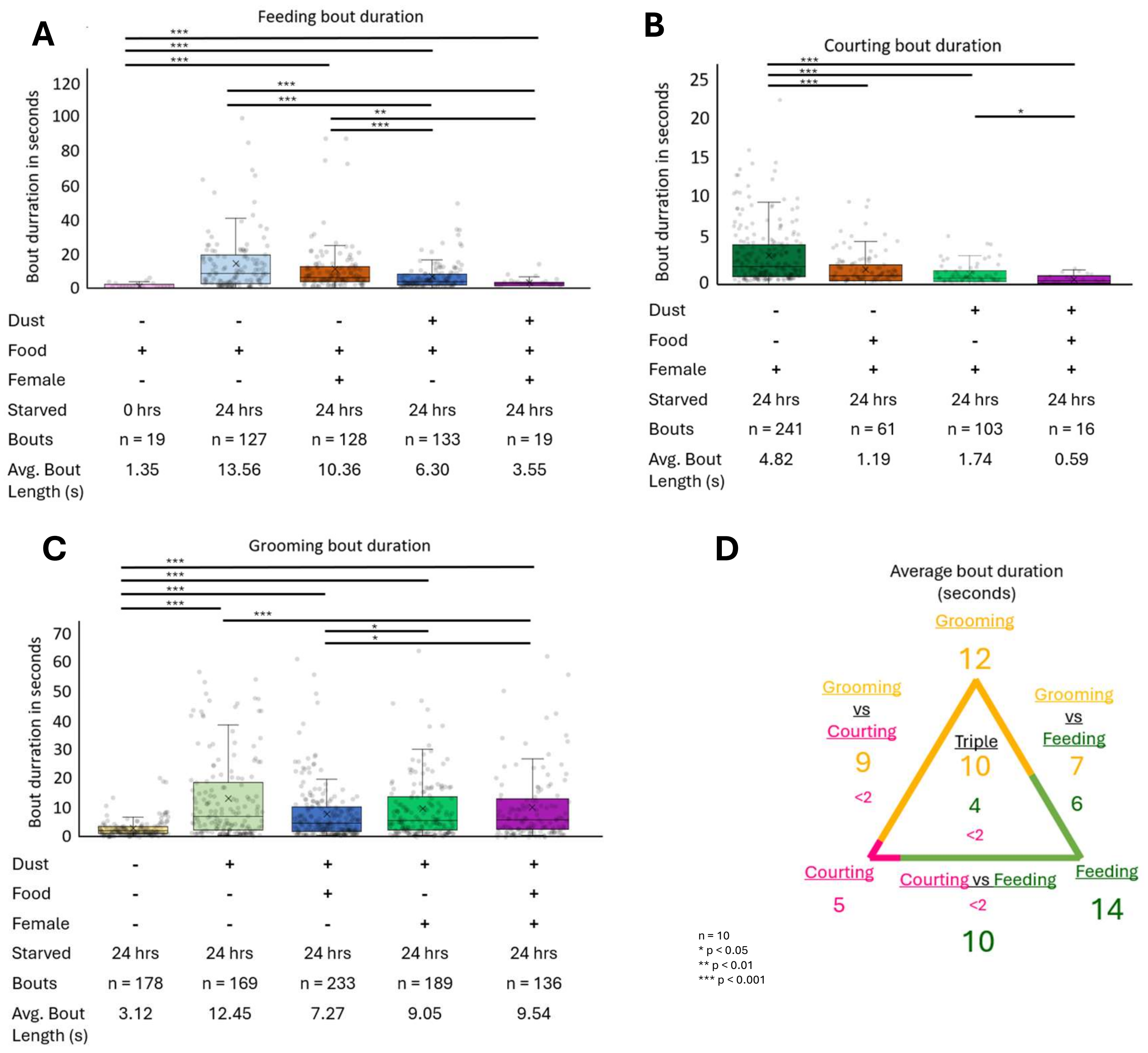

Fig. S2. Effect of conflicting choice on bouts of behavior.

A) Box plot with a dot plot overlay depicting individual feeding bout lengths from ten flies each within five minutes for each condition. B) Box plot with dot plot overlay depicting courting bouts. This plot excludes orienting, a non-active step in courtship. C) Box plot with dot plot overlay depicting grooming bouts. Outliers are not shown in the graph. D) A summary triangle showing average bout duration in seconds under the dusted and starved (grooming); food and starved (feeding); virgin female and starved (courting); dusted, starved, and food (grooming vs feeding); dusted, starved, and virgin female (grooming vs courting); starved, food, and virgin female (courting vs feeding); and starved, dusted, food, and virgin female (triple) conditions. In the box plots, X denotes mean, bar denotes median, and whiskers denote the lower and upper quartile. Statistics were obtained using a Kruskal-Wallis test followed by a Dunn's multiple comparison test and Bonferroni correction.

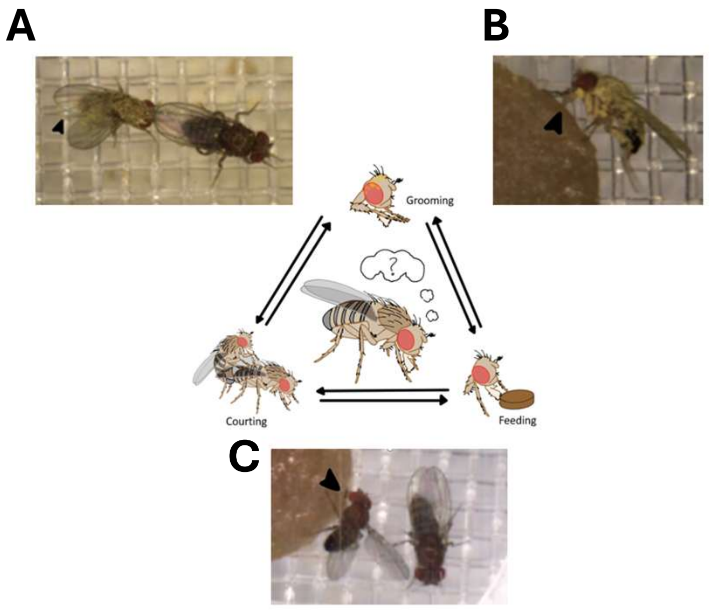

Fig. S3. Simultaneous behaviors in competition assays.
A) A male fly simultaneously wing singing and back leg grooming (arrow). B) A male fly simultaneously feeding (arrow) and back leg grooming. C) A male fly simultaneously feeding (arrow) and wing singing.

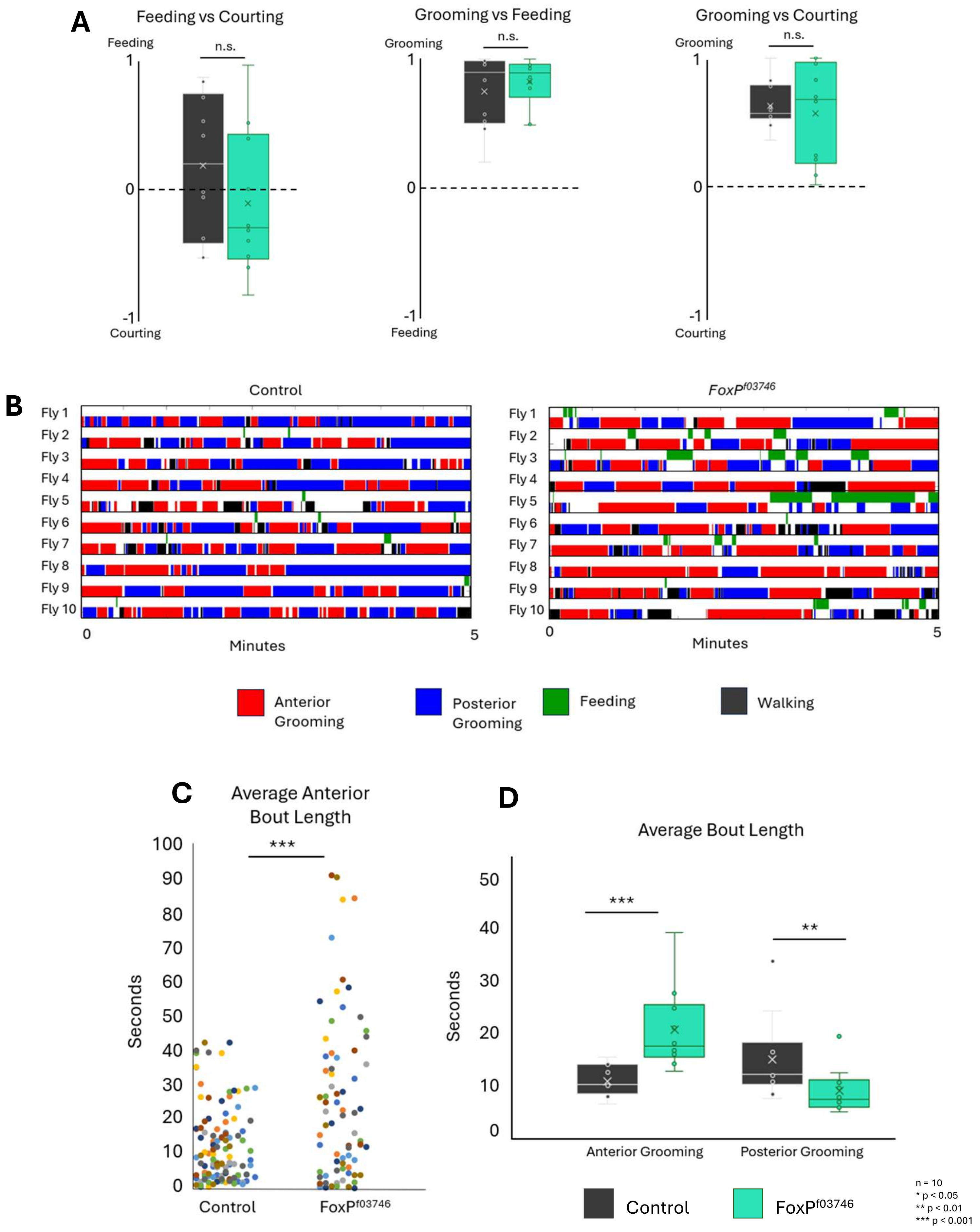
A
Feeding vs Courting
Feeding
Courting
n.s.
Grooming vs Feeding
Grooming
Feeding
n.s.
Grooming vs Courting
Grooming
Courting
n.s.
B
Control
FoxP^f03746
Fly 1
Fly 2
Fly 3
Fly 4
Fly 5
Fly 6
Fly 7
Fly 8
Fly 9
Fly 10
Minutes
Anterior Grooming
Posterior Grooming
Feeding
Walking
C
Average Anterior Bout Length
Seconds
***
Control
FoxP^f03746
D
Average Bout Length
Seconds
***
**
Anterior Grooming
Posterior Grooming
Control
FoxP^f03746
n = 10
* p < 0.05
** p < 0.01
*** p < 0.001

Fig. S4. *FoxP* mutants show a change in type of grooming behaviors compared to a *w1118* control.

A) Preference index of a *FoxP* mutant during the three competition assays. B) Ethograms of the first five minutes of a grooming vs feeding assay. C) Dot plot depicting anterior grooming bouts in a five-minute period with dusted flies. Each color is a different fly. n = 10 flies. D) Box plot comparing anterior and posterior grooming in dusted flies. In the box plots, the X denotes mean, bar denotes median, and whiskers denote the lower and upper quartile. Statistics were obtained using a Mann-Whitney test. All figures are n = 10.

Journal of Experimental Biology • Supplementary information

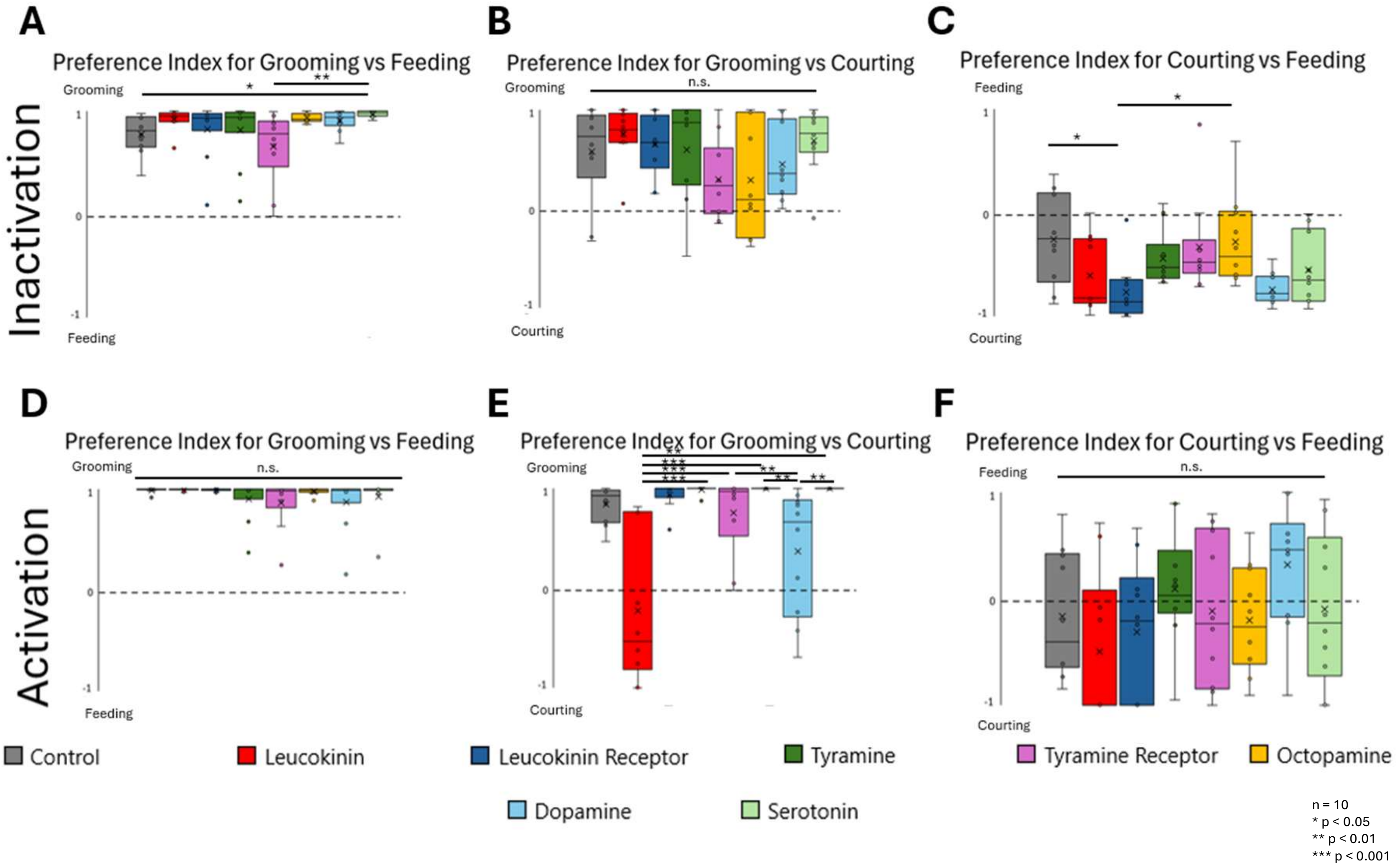

Fig. S5. Screen results of activating and inactivating different neuron subsets.
A) Preference index for grooming vs feeding when inactivating neurons subsets. B) Preference index for grooming vs courting when inactivating neurons subsets. C) Preference index for courting vs feeding when inactivating neurons subsets. D) Preference index for grooming vs feeding when activating neurons subsets. E) Preference index for grooming vs courting when activating neurons subsets. F) Preference index for courting vs feeding when activating neurons subsets. Statsiticas were obtained using a Kruskal-Wallis test followed by a Dunn's multiple comparison test and Bonferroni correction.

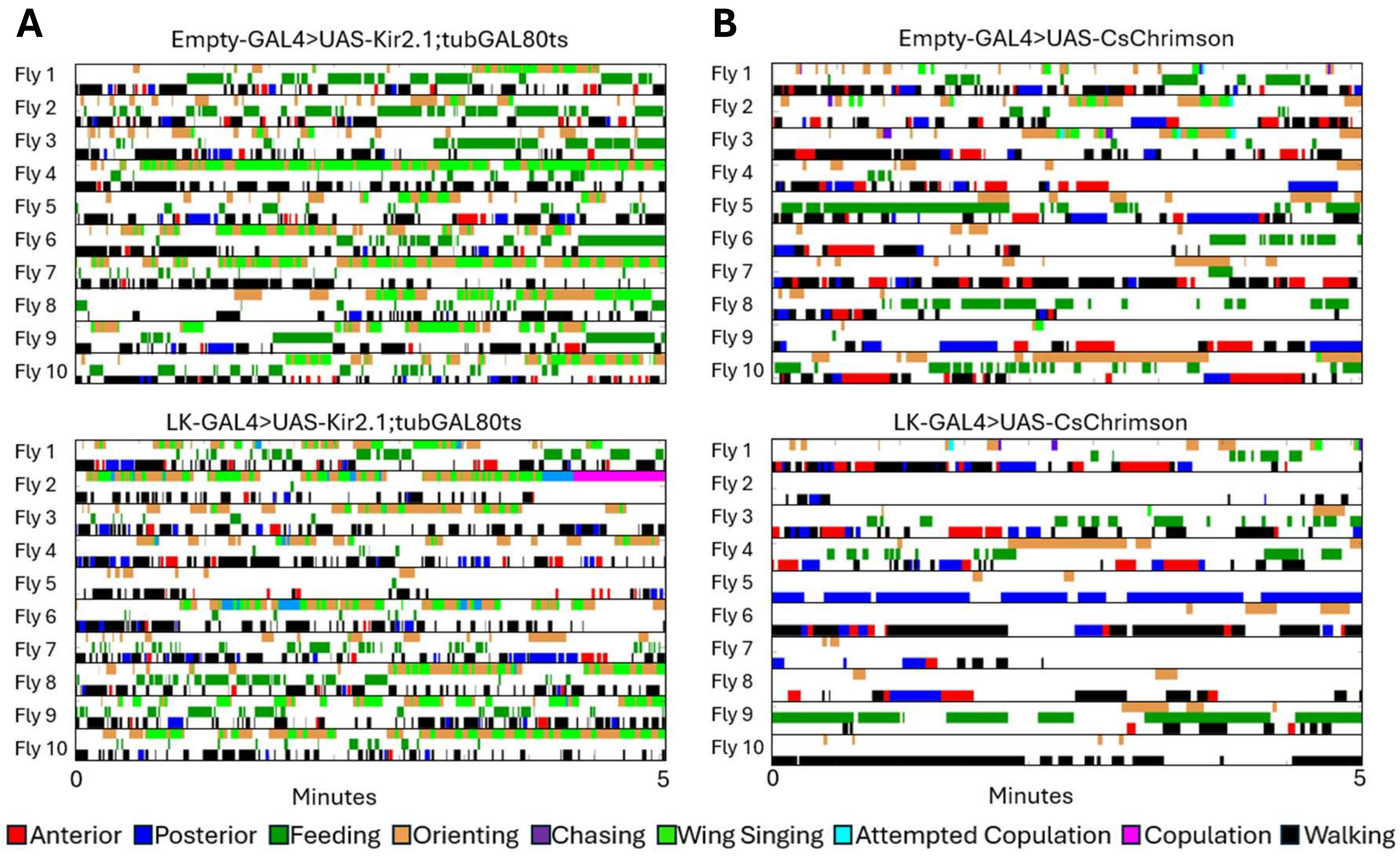

Fig. S6. Examples of ethogram analysis of individual components of the grooming, feeding, and courting behaviors.

A) Ethograms depicting feeding vs courting behavior when inactivating leucokinin producing neurons. Grooming and courting behaviors are broken into individual component behavior sequences. B) Ethograms depicting feeding vs courting behavior when activating leucokinin producing neurons.

Journal of Experimental Biology • Supplementary information
